# Increased complement activation is a distinctive feature of severe SARS-CoV-2 infection

**DOI:** 10.1126/sciimmunol.abh2259

**Published:** 2021-05-13

**Authors:** Lina Ma, Sanjaya K. Sahu, Marlene Cano, Vasanthan Kuppuswamy, Jamal Bajwa, Ja’Nia McPhatter, Alexander Pine, Matthew L. Meizlish, George Goshua, C-Hong Chang, Hanming Zhang, Christina Price, Parveen Bahel, Henry Rinder, Tingting Lei, Aaron Day, Daniel Reynolds, Xiaobo Wu, Rebecca Schriefer, Adriana M. Rauseo, Charles W. Goss, Jane A. O’Halloran, Rachel M. Presti, Alfred H. Kim, Andrew E. Gelman, Charles S. Dela Cruz, Alfred I. Lee, Philip A. Mudd, Hyung J. Chun, John P. Atkinson, Hrishikesh S. Kulkarni

**Affiliations:** 1 Division of Pulmonary and Critical Care Medicine, John T. Milliken Department of Medicine, Washington University School of Medicine; St. Louis, USA.; 2 Division of Hospital Medicine, John T. Milliken Department of Medicine, Washington University School of Medicine; St. Louis, USA.; 3 Marian University; Indianapolis, USA.; 4 University of Pittsburgh; Pittsburgh, USA.; 5 Section of Hematology, Department of Internal Medicine, Yale School of Medicine; New Haven, USA.; 6 Yale School of Medicine; New Haven, USA.; 7 Yale Cardiovascular Research Center, Section of Cardiovascular Medicine, Department of Internal Medicine, Yale School of Medicine; New Haven, USA.; 8 Section of Immunology, Department of Internal Medicine, Yale School of Medicine; New Haven, USA.; 9 Yale New Haven Health System; New Haven, USA.; 10 Department of Pathology and Immunology, Washington University School of Medicine; St. Louis, USA.; 11 Department of Emergency Medicine, Washington University School of Medicine; St. Louis, USA.; 12 Division of Rheumatology, John T. Milliken Department of Medicine, Washington University School of Medicine; St. Louis, USA.; 13 Division of Infectious Diseases, John T. Milliken Department of Medicine, Washington University School of Medicine; St. Louis, USA.; 14 Division of Biostatistics, Washington University School of Medicine; St. Louis, USA.; 15 Section of Pulmonary, Critical Care, and Sleep Medicine, Department of Internal Medicine, Yale School of Medicine; New Haven, USA.

## Abstract

Complement activation has been implicated in the pathogenesis of severe SARS-CoV-2 infection. However, it remains to be determined whether increased complement activation is a broad indicator of critical illness (and thus, no different in COVID-19). It is also unclear which pathways are contributing to complement activation in COVID-19, and if complement activation is associated with certain features of severe SARS-CoV-2 infection, such as endothelial injury and hypercoagulability. To address these questions, we investigated complement activation in the plasma from patients with COVID-19 prospectively enrolled at two tertiary care centers: Washington University School of Medicine (n=134) and Yale School of Medicine (n=49). We compared our patients to two non-COVID cohorts: (a) patients hospitalized with influenza (n=54), and (b) patients admitted to the intensive care unit (ICU) with acute respiratory failure requiring invasive mechanical ventilation (IMV, n=22). We demonstrate that circulating markers of complement activation are elevated in patients with COVID-19 compared to those with influenza and to patients with non-COVID-19 respiratory failure. Further, the results facilitate distinguishing those who are at higher risk of worse outcomes such as requiring ICU admission, or IMV. Moreover, the results indicate enhanced activation of the alternative complement pathway is most prevalent in patients with severe COVID-19 and is associated with markers of endothelial injury (i.e., angiopoietin-2) as well as hypercoagulability (i.e., thrombomodulin and von Willebrand factor). Our findings identify complement activation to be a distinctive feature of COVID-19, and provide specific targets that may be utilized for risk prognostication, drug discovery and personalized clinical trials.

## Teaser

Increased complement activation is observed in COVID-19 compared to non-COVID respiratory illness, and is a key marker of organ failure.

## INTRODUCTION

Morbidity and mortality associated with SARS-CoV-2 infection (i.e., COVID-19) have been attributed to a hyperinflammatory phase (*1*–*4*). Specifically, approximately 7-10 days after clinical onset, a subset of patients require hospitalization, ICU admission, and mechanical ventilation, and may ultimately die due to their illness (*5*). However, the components of the immune response that contribute to critical illness in COVID-19 remain incompletely understood. For example, although certain cytokines such as IL-6, G-CSF, IL-1RA, and MCP-1 predict death in COVID-19, their circulating levels are no different when measured in patients with other viral infections, such as influenza (*6*). However, the clinical presentation and autopsy findings of patients with COVID-19 indicate that in at least some of these patients, there may be a distinct immunological response, which is responsible for the mortality rate in excess of other viral illnesses (i.e., influenza) (*7*, *8*), and results in certain coagulopathic events such as microscopic and macroscopic thrombi occurring more commonly in COVID-19 (*9*–*13*). Understanding the underlying mechanisms of these relatively unique aspects of COVID-19 is crucial for targeting therapies, and, may provide insights into the pathogenesis of acute respiratory distress syndrome on a broader scale.

The complement system, one of the first lines of the host defense and a key player in the innate immune response, has been implicated in the pathogenesis of severe COVID-19 (*14*–*16*). Features of disease such as hypercoagulability and tissue necrosis, as well as genetic factors have increased the suspicion that the complement system contributes to severe illness (*14*, *17*–*19*). The system can be activated by three arms – the classical, lectin or the alternative pathway (*20*). Although a prevailing hypothesis is that the N-protein of coronaviruses triggers MASP2-mediated complement activation and thus drives disease severity via the lectin pathway (*21*), in vitro studies have suggested that spike proteins (subunits S1 and S2) of SARS-CoV-2 activate the alternative pathway (*22*). Regardless of the initial activation step, the system converges on the cleavage of C3 and subsequently C5 to anaphylatoxins that facilitate vasodilation, chemotaxis and thrombosis (C3a, C5a). Further, activation of the system facilitates opsonization (via C3b), and membrane attack complex formation (MAC, i.e., C5b-9) (*20*). Accordingly, multiple studies have demonstrated elevation of C5a and sC5b-9 in patients with COVID-19 (*15*, *23*, *24*), as well as deposition of activated complement proteins in injured tissues and organs (*25*, *26*). As a result, these studies have created a precedent for targeting the complement system in multiple ongoing phase II and phase III clinical trials employing complement inhibitors in COVID-19 (*27*–*30*).

Most studies addressing the role of complement in COVID-19 have not included acute respiratory infection cohorts without COVID-19. Therefore, it is unclear whether complement activation is distinctive feature of severe COVID-19, or simply a broader feature of critical illness. Additionally, which arms of the complement cascade contribute to complement activation in patients with COVID-19, remains to be defined. Finally, whether complement activation in vivo is associated with certain distinctive features of COVID-19 (i.e., endothelial injury and hypercoagulability) is also unclear. Here we report that increased complement activation is an immunological feature of COVID-19, which distinguishes those developing severe illness. Using two independent cohorts, we identify components of the alternative pathway are markedly elevated in patients with severe COVID-19. Our findings may potentially refine the approach for therapeutically targeting the complement system in severe SARS-CoV-2-infection.

## RESULTS

### Markers of complement activation are higher in COVID-19 compared to non-COVID-19 respiratory failure

**Fig. 1:**
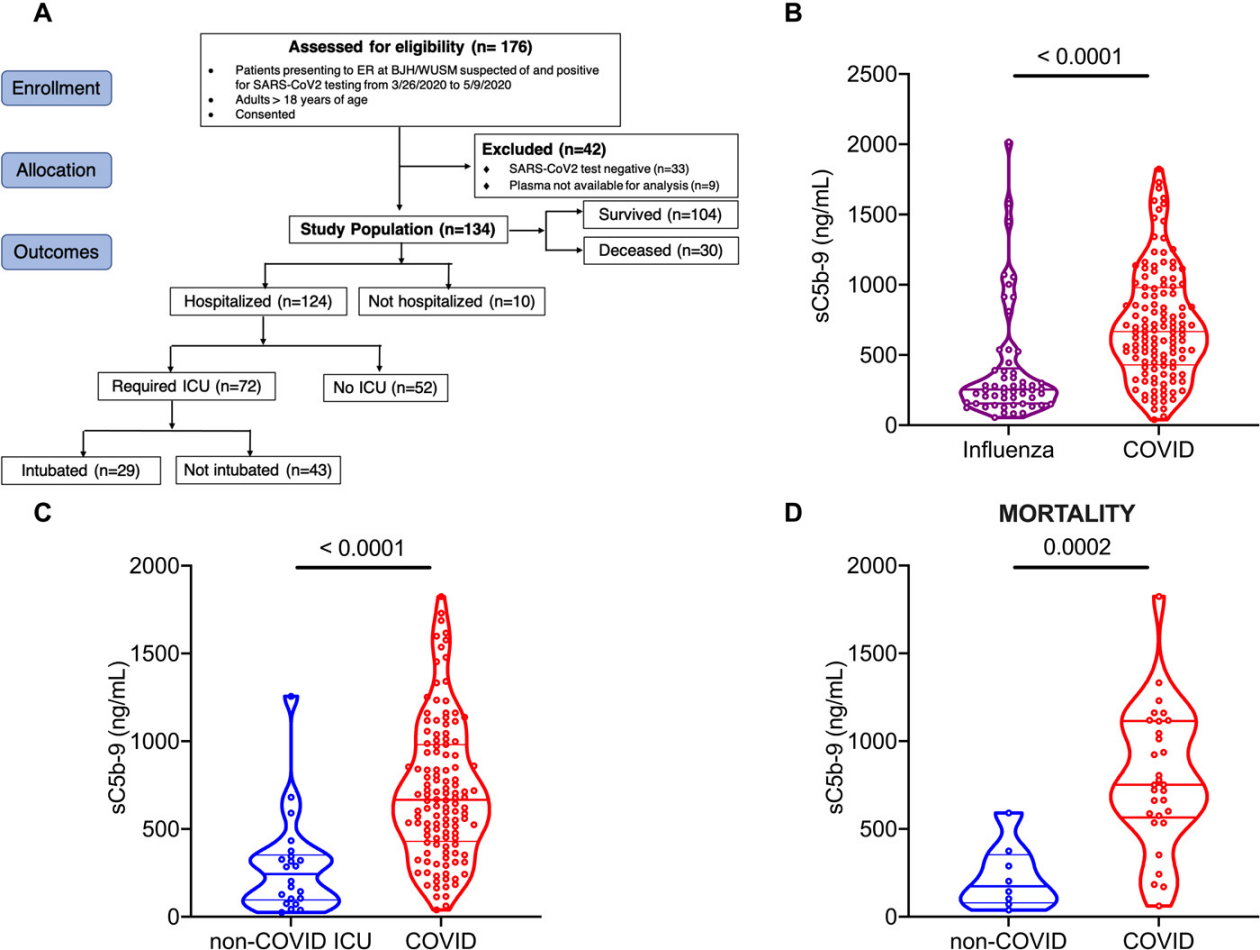
Markers of complement activation are higher in COVID-19 compared to non-COVID-19 respiratory failure. Plasma for determination of circulating markers of complement activation was obtained in patients with COVID-19 (n=134) and influenza (n=54) at Barnes-Jewish Hospital (BJH)/Washington University School of Medicine (WUSM). (A) CONSORT flow diagram showing patient enrollment, allocation and outcomes in the COVID-19 cohort. The CONSORT diagram for the influenza and non-COVID acute respiratory failure cohorts are in Figure S1. Violin plots of differences in sC5b-9 between (B) the influenza (EDFLU, n=54) and COVID-19 (n=124) cohorts, (C) the non-COVID acute respiratory failure (Immunity in Pneumonia and Sepsis, IPS, n=22) and the COVID-19 cohorts (n=124), and (D) restricting both the cohorts from Fig. 1C to those who died (non-COVID, IPS, n=8 vs COVID-19, n=30). Statistical significance is determined using Mann-Whitney U test.

We first sought to assess complement activation in patients hospitalized with COVID-19, versus those with non-COVID-19-related illness. We compared plasma sC5b-9 levels in 134 patients with COVID-19 at WUSM (**Table 1**, **Fig. 1A****)** — with two independent cohorts of non-COVID-19 acute respiratory illnesses (**Table 1****, Figures S1A and S1B**). The first comparison was with the EDFLU cohort of 54 patients presenting with influenza at WUSM. Patients hospitalized with COVID-19 had significantly higher median plasma sC5b-9 levels [666.3 (interquartile range, 429.7 – 980.1) ng/mL] compared to those with influenza [254.5 (154.5 – 403.8) ng/mL, p<0.0001, **Fig. 1B**]. Given that a minority of the influenza cohort required invasive mechanical ventilation (IMV, 6/54, 11%), we also compared the plasma sC5b-9 levels of patients in our COVID-19 cohort with patients in our IPS (Immunity in Pneumonia and Sepsis) cohort, all of whom required IMV in the ICU for non-COVID-19-related acute respiratory failure (N=22). Plasma sC5b-9 levels were higher in patients with COVID-19 when compared to the IPS cohort [243.5 (95.62 – 352.1) ng/mL, p<0.0001, **Fig. 1C**]. Plasma sC5b-9 levels in the COVID-19 cohort remained higher than the levels in the IPS cohort despite restricting the COVID-19 cohort to those admitted to the ICU (**Figure S1C)** and among those requiring IMV (**Figure S1D**). These observations suggested that patients with COVID-19 appear to have higher circulating markers of complement activation compared to patients with non-COVID-19-related acute respiratory infection. When restricted to those who died, patients in the COVID-19 cohort had higher plasma sC5b-9 levels [751.7 (575.2 – 1,118) ng/mL, N=31] compared to the non-COVID-19 IPS cohort [173.3 (78.99 – 353.3) ng/mL, N=8, p < 0.0001, **Fig. 1D**].

**Table 1:** Demographic characteristics of the cohorts.

	**COVID (n=134)**	**EDFLU (n=54)**	**ISF (n=22)**	**Yale, LT cohort (n=23)**	**Yale, CS cohort (n=49)**
**Demographics**					
Age in years, mean +/− SD	63 ± 16	53 ± 17	54 ± 17	65 ± 12	63 ± 17
**Gender**					
Female	41% (55)	50% (27)	50% (11)	39% (9)	33% (16)
Male	59% (79)	50% (27)	50% (11)	61% (14)	67% (33)
**Ethnicity**					
Black or African American	79.1% (106)		54.5% (12)	26% (6)	24.5% (12)
White	19.4% (26)		40.9% (9)	52% (12)	51% (25)
Other	1.5% (2)		4.5% (1)	21% (5)^c^	24.5% (12)^c^
**Clinical Characteristics**					
Hospital Admission	92.5% (124)	96.3% (52)	100.0% (22)	100% (23)	100% (49)
ICU Admission	53.7% (72)	24.1% (13)	100.0% (22)	61% (14)	82% (40)
IMV	21.6% (29)	11.1% (6)	100.0% (22)	9% (2)	53% (26)
In-Hospital Mortality	22.4% (30)	3.7% (2)	36.4% (8)	30% (7)	24.5 (12)
**Comorbidities**					
Smoking History^a^	46.3% (62)	-	63.6% (14)	65% (15)	8% (4)
Chronic Lung Disease	19.4% (26)	22.2% (12)	13.6% (3)	35% (8)	10% (5)
End-stage renal disease (ESRD)^b^	6% (8)	7.4% (4)	4.5% (1)	4% (1)	4% (2)
Diabetes mellitus, type 2	52.2% (70)	35.2% (19)	18.2% (4)	39% (9)	27% (13)

Abbreviations: COVID-19: subjects with SARS-CoV-2 infection; CS: cross-sectional; ICU: intensive care unit; EDFLU: Barnes Jewish Hospital Emergency Department Influenza; ISF: Immunity in Pneumonia and Sepsis; LT: longitudinal; SD: standard deviation.

^a^ Current or former smoker. Smoking history was unavailable for the EDFLU cohort.

^b^ Based on chronic dialysis requirement.

^c^ Hispanic/Latino, Asian, and unknown.

### Complement activation is associated with worse outcomes in two independent COVID-19 cohorts

**Fig. 2:**
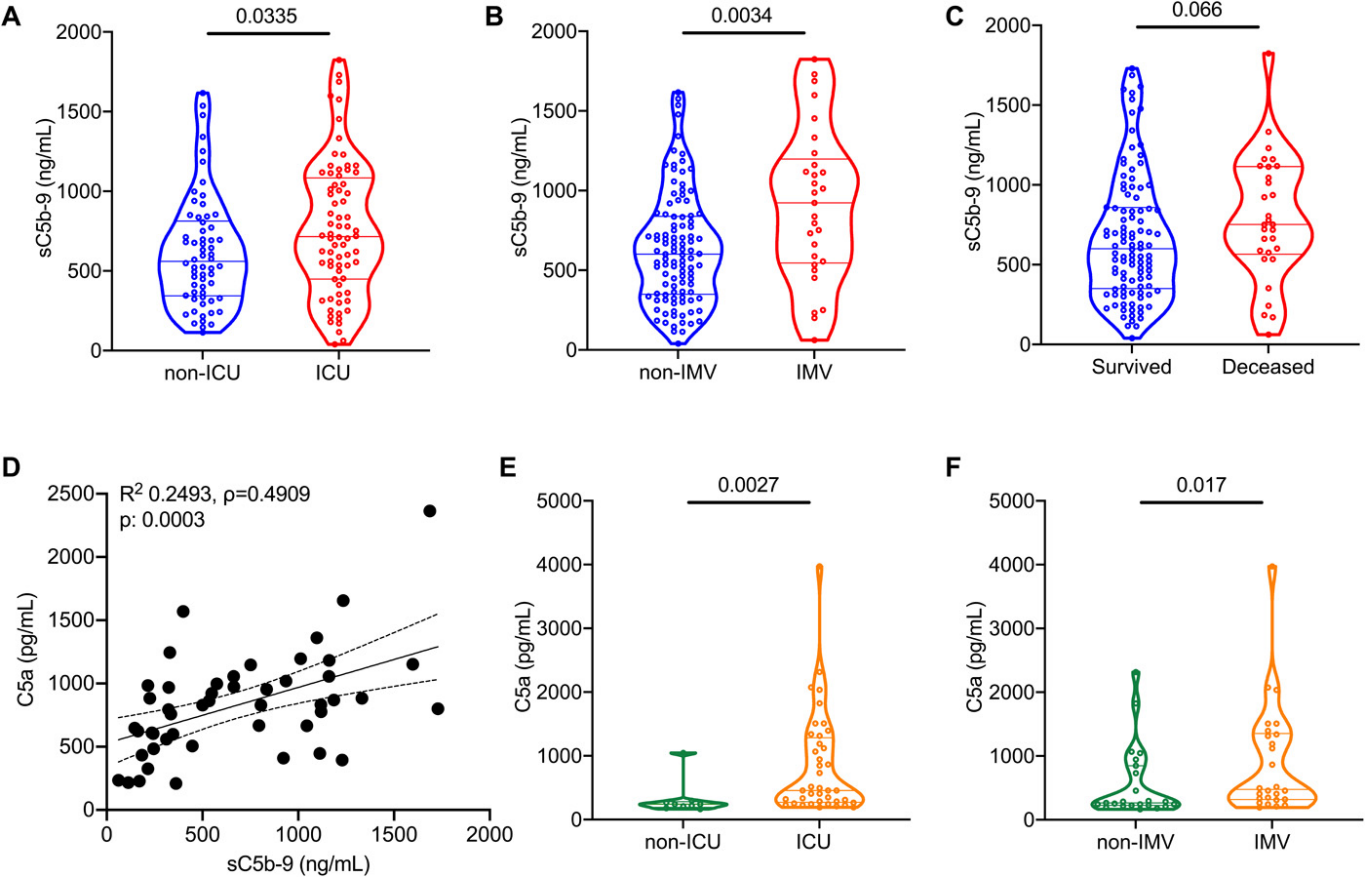
Complement activation is associated with worse outcomes in COVID-19 in two independent cohorts. Markers of complement activation were quantified in the plasma at WUSM and Yale University School of Medicine (Yale). Violin plots of sC5b-9 levels in the WUSM COVID-19 cohort in (A) patients requiring ICU admission (n=72) versus those who did not (n=62), (B) patients requiring invasive mechanical ventilation (IMV, n=29) versus those who did not (n=105), and (C) patients who died (n=30) versus those who survived (n=104). (D) A linear regression line shows the relationship between plasma levels of sC5b-9 and C5a. The spline chart demonstrates the mean with 95% confidence intervals. R^2^ represents the goodness-of-fit. The degree of correlation is assessed using Spearman’s rank correlation coefficient test (ρ=0.4909, 95% CI 0.2321 – 0.6848, N=48). In the Yale cross-sectional cohort, concurrently measured C5a levels are utilized to compare (E) patients requiring ICU admission (n=40) versus those who did not (n=9), and (F) patients requiring IMV (n=26) versus those who did not (n=23). Statistical significance is determined using Mann-Whitney U test.

Plasma sC5b-9 levels were higher in patients belonging to the WUSM COVID-19 cohort who required hospitalization [666.3 (429.7 – 980.1) ng/mL, N=124], compared to those who were discharged from the emergency room [326.2 (211.6 – 584.4) ng/mL, N=10, p=0.0097, **Figure S2A**], as well as in those requiring ICU admission (**Table 2**, **Fig. 2A**). Plasma sC5b-9 levels were higher in patients who required IMV [922.8 (545.0 – 1,198.0) ng/mL, N=29] compared to those who did not [600.8 (349.2 – 838.8) ng/mL, N=105, p=0.0034, **Fig. 2B**]. This comparison held even when restricting the cohort to those who were hospitalized, when comparing those on IMV versus those not on IMV (**Figure S2B**), and when restricting the cohort to those who were admitted to the ICU, when comparing those on IMV versus those not on IMV (**Figure S2C**). Patients with COVID-19 who died had higher plasma sC5b-9 levels [751.4 (565.2 – 1,115) ng/mL, N=30] compared to those who survived the index hospitalization, although this did not reach statistical significance [600.0 (349.9 – 858.5) ng/mL, N=104, p=0.0666, **Fig. 2C**]. We also measured C5a, which is a product of C5 cleavage similar to C5b (that contributes to the formation of membrane attack complex, C5b-9). In the WUSM cohort, plasma C5a correlated with sC5b-9 (ρ=0.4909, **Fig. 2D**), and C5a levels were significantly higher in patients with COVID-19 requiring ICU admission compared to those who did not (**Table 2**, **Figure S2D**).

**Table 2:** Complement analytes in the Washington University School of Medicine (WUSM) COVID-19 cohort.

	**Non-ICU (N=62)**	**ICU (N=72)**	**p**
sC5b-9 (ng/mL)	559.5 (343.3 – 813.0)	715.4 (448.5 – 1,084.0)	0.0335
C5a (pg/mL)*	635.0 (471.9 – 892.6)	918.4 (666.7 – 1,081)	0.034
iC3b: C3 ratio*	0.56 (0.51 – 0.65)	0.70 (0.57 – 1.39)	0.002
Factor B, ng/mL*	21,606 (17,834 – 26,853)	25,840 (20,544 – 32,832)	0.033
AP hemolytic activity, %**	85.0 (76.0 – 102.5)	81.0 (74.5 – 90.0)	0.2519
Ba, ng/mL*	1,191 (901.3 – 1,981)	3,112 (2,022-6,612)	<0.0001
Factor D, ng/mL*	4,640.3 (3,659 – 9,887.3)	6,622.5 (4,308 – 10,854.1)	0.166

*C5a, iC3b: C3 ratio, Factor B, Ba and Factor D were measured in 48 patients, among whom 26 needed an intensive care unit (ICU) admission and 22 did not. **Alternative pathway (AP) hemolytic activity was performed in 38 patients (21 ICU, 17 non-ICU) based on the availability of samples. Statistical tests for comparison were done using the Mann-Whitney U test. Values are represented as median (interquartile range).

To test whether our findings hold true at a center that had independently measured inflammatory markers in a similar time frame of patient enrollment, we utilized a second cohort from Yale School of Medicine, wherein C5a had been prospectively measured in the plasma of patients hospitalized with COVID-19 (Yale longitudinal cohort, N=23) within the first 24 hours of hospital admission. In this cohort, plasma C5a levels were significantly higher in those patients who requiring ICU admission (**Table 3****, Figure S2E**). In this cohort, there were not enough patients to make a meaningful comparison regarding the need for IMV (N=2, 9%). Hence, we expanded the cohort to include those patients who had their first plasma sampled beyond the first day of hospital admission (Yale cross-sectional cohort, N=49). Even in this expanded cohort, plasma C5a levels remained significantly higher in hospitalized patients with COVID-19 requiring ICU admission [456.9 (269.2 – 1,282) pg/mL, N=40] versus those who did not [243.9 (193.3 – 280.3) pg/mL, N=9, p=0.0027, **Fig. 2E**]. Additionally, among those patients with COVID-19 who were hospitalized, plasma C5a levels were significantly higher in those requiring IMV (**Table 4**, **Fig. 2F**).

**Table 3:** Complement analytes in patients with COVID-19 in the Yale School of Medicine longitudinal cohort.

	**Non-ICU (N=14)**	**ICU (N=9)**	**p**
C5a, pg/mL	43.2 (43.2 – 43.2)	77.6 (43.2 – 285.6)	0.0016
Factor D, ng/mL	1,442 (1,234 – 1,803)	1,825 (1,541 – 2,576)	0.07

Abbreviations: ICU: intensive care unit. Statistical tests for comparison were done using the Mann-Whitney U test. All samples drawn within 24 hours of hospital admission. Values are represented as median (interquartile range).

**Table 4:** Markers of complement activation, endothelial injury and coagulation in the Yale School of Medicine cross-sectional cohort.

	**Non-IMV (N=23)**	**IMV (N=26)**	**p**
C5a, pg/mL	263.8 (225.5 – 848)	475.6 (317.9 – 1353.0)	0.017
Factor D, ng/mL	4, 605 (3,721 – 6,187)	6,437 (3,445 – 9,674)	0.09
Ang2, ng/mL	4,077 (2,149 – 7,633)	11,470 (6,711 – 15,103)	<0.0001
Thrombomodulin, ng/mL	2.9 (1.9 – 4.5)	5.0 (3.0 – 8.1)	0.0068
vWF:Ag, %	375.0 (266.0 – 559.0)*	558.5 (409.8 – 685.3)	0.0063

Abbreviations: Ang2: Angiopoietin-2, vWF:Ag: von Willebrand factor antigen. *Samples for measuring vWF:Ag were available in 21 out of 23 patients who did not need IMV. Statistical tests for comparison were done using the Mann-Whitney U test. Values are represented as median (interquartile range).

### Increase in components of the alternative pathway are associated with worse outcomes in COVID-19

**Fig. 3:**
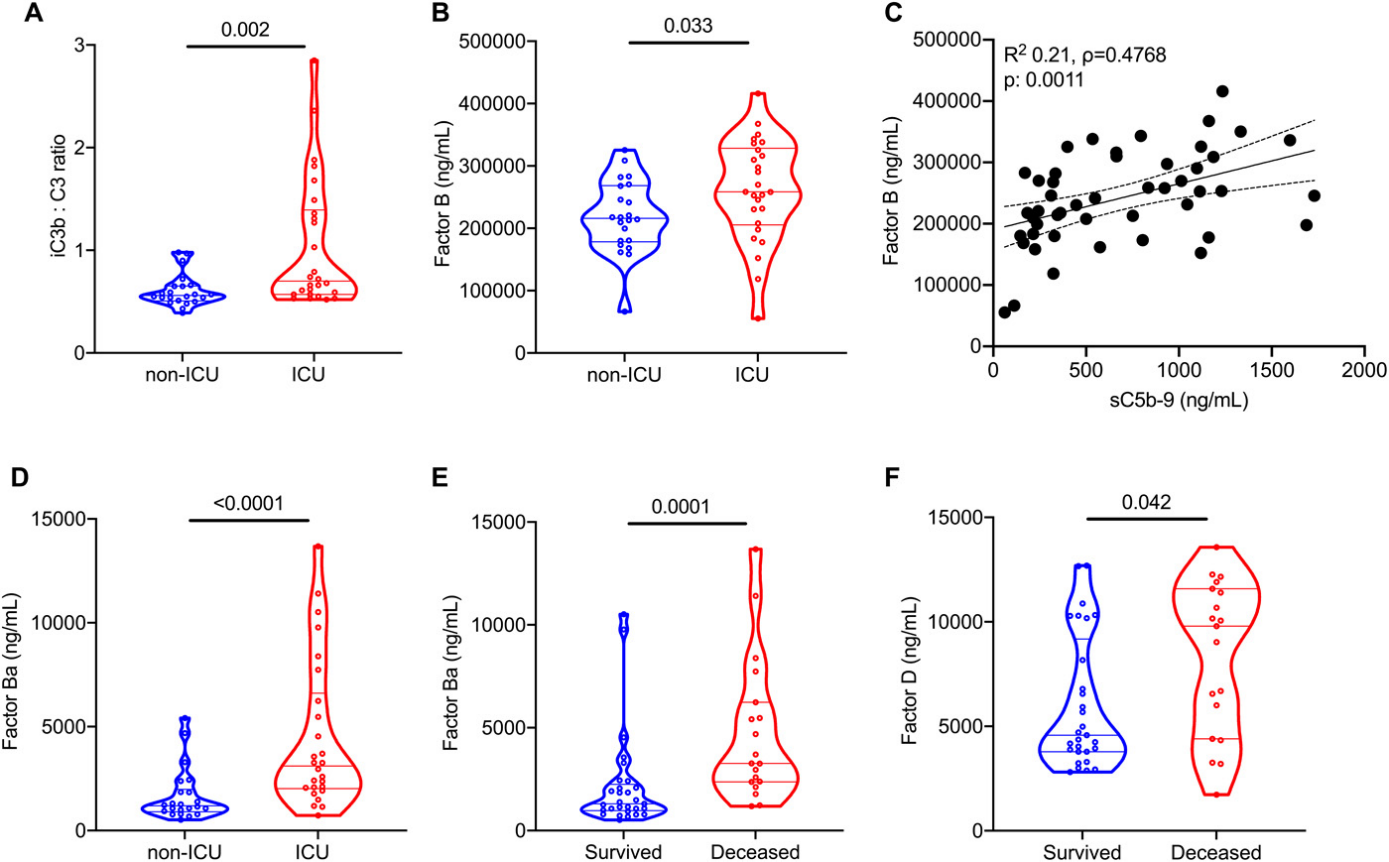
Alternative pathway activation is associated with worse outcomes in COVID-19. Comparisons in the levels of components involved in the alternative pathway (AP) in plasma of patients requiring ICU admission (n=26) versus those who did not (n=22), in the WUSM COVID-19 cohort, are presented using violin plots - (A) iC3b: C3 ratio, (B) Factor B, and (D) Ba. (C) A linear regression line shows the relationship between plasma levels of sC5b-9 and Factor B. The spline chart demonstrates the mean with 95% confidence intervals. R^2^ represents the goodness-of-fit. The degree of correlation is assessed using Spearman’s rank correlation coefficient test (ρ=0.4768, 95% CI 0.2146 – 0.6749, N=48). (E) Plasma Ba levels are compared in patients who survived [1,301.0 (966.0 – 2250.0), N=29] versus those who did not [3,266 (2,368 – 6236), N=19], as are the plasma levels of Factor D (F). Statistical significance is determined using Mann-Whitney U test.

We also investigated specific components of the complement cascade that may facilitate complement activation in COVID-19. In the WUSM cohort, the ratio of iC3b: C3 levels, which indicates complement activation resulting in cleavage of C3 (and is suggestive of but is not exclusively restricted to alternative pathway activation), was higher in patients needing ICU admission (**Table 2****,** **Fig. 3A**), including those requiring IMV versus those who did not (**Figure S3A**). Of note, Factor B, a component of the alternative pathway, was increased in patients with COVID-19 requiring ICU admission (**Table 2****,** **Fig. 3B**). Factor B levels also correlated with sC5b-9 levels (ρ=0.4768, **Fig. 3C**). Levels of Ba, which reflect activation of the alternative pathway, were significantly higher in patients with COVID-19 who required ICU admission (**Table 2****,** **Fig. 3D**), as well as those requiring IMV (**Figure S3B**), and those who did not survive the initial hospitalization (**Fig. 3E**). The alternative pathway hemolytic activity was preserved in the COVID-19 cohort (**Table 2****, Figure S3C**). Factor D was significantly higher in those who died [9,791 (4,400 – 11,579) ng/mL, N=19] compared to those who survived [4,572 (3,784 – 9,175) ng/mL, N=29, p=0.042, **Fig. 3F**]. Although Factor D did not distinguish those patients requiring IMV (**Figure S3D**), it trended higher in those who required renal replacement therapy [RRT, 10,158 (4,432 – 12,422) ng/mL, N=9] versus those who did not [4,983 (3,786 – 10,176) ng/mL, N=39, p=0.08, **Figure S3E**]. Plasma Factor D levels of patients with COVID-19 requiring ICU admission were higher than those who did not in the Yale cohort (**Table 3**, **Figure S3F).**

### Complement activation is associated with markers of endothelial injury and a prothrombotic state in patients with COVID-19

**Fig. 4:**
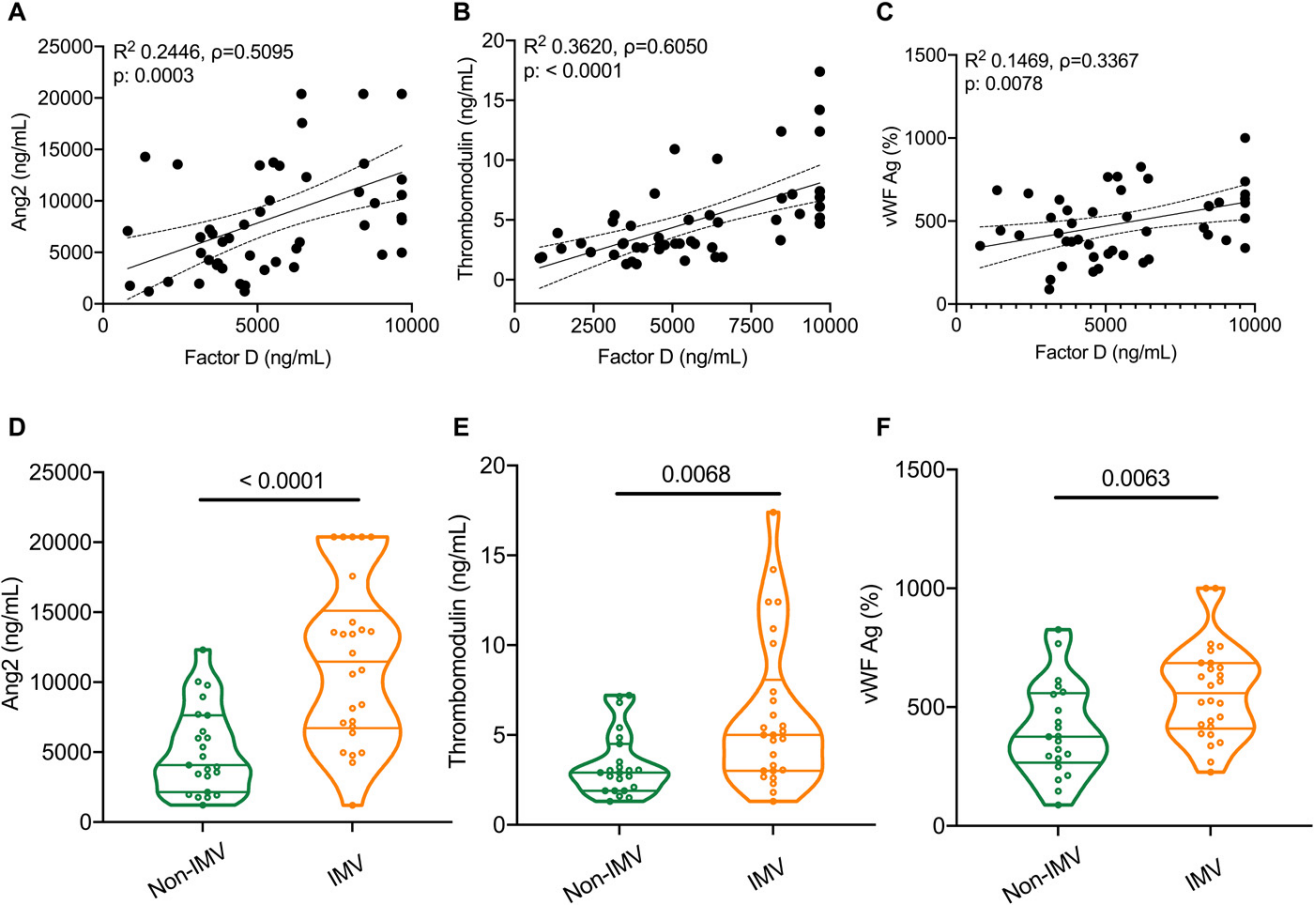
Complement activation is associated with markers of endothelial injury and a prothrombotic state in patients with COVID-19. A linear regression line shows the relationship between plasma levels of Factor D and (A) angiopoietin-2 (Ang2), (B) thrombomodulin, and (C) von Willebrand factor antigen (vWF:Ag) in the Yale cross-sectional cohort. The spline chart demonstrates the mean with 95% confidence intervals. R^2^ represents the goodness-of-fit. The degree of correlation is assessed using Spearman’s rank correlation coefficient test between Factor D and (a) Ang2 (ρ=0.5095, 95% CI 0.2585 – 0.6960, n=49), (b) thrombomodulin (ρ=0.6050, 95% CI 0.3829 – 0.7609, n=49), and (c) vWF:Ag (ρ=0.3367, 95% CI 0.04612 – 0.5747, n=47). Violin plots are utilized for comparing the levels of (D) Ang2, (E) thrombomodulin, and (F) vWF:Ag in plasma of patients requiring invasive mechanical ventilation (IMV) versus those who did not. Statistical significance is determined using Mann-Whitney U test.

Complement activation has primarily being implicated in multiorgan failure in COVID-19 due to its role in endothelial injury and inducing a prothrombotic state. Consequently, we investigated the association between Factor D and commonly utilized markers of endothelial injury, angiopoietin-2 (Ang2), and a prothrombotic state, namely thrombomodulin and the von Willebrand factor antigen (vWF:Ag). Factor D strongly correlated with Ang2 (ρ=0.5095, **Fig. 4A**) and thrombomodulin (ρ=0.6050, **Fig. 4B**). There was a modest correlation between Factor D and vWF:Ag (ρ=0.3367, **Fig. 4C**). Ang2 was significantly higher in ICU patients with COVID-19 requiring IMV compared to those who did not (**Table 4**, **Fig. 4D**), as was thrombomodulin (**Table 4****,** **Fig. 4E****)** and vWF:Ag (**Table 4**, **Fig. 4F****).**

## DISCUSSION

The complement system has been implicated in COVID-19 since early in the pandemic, as evidenced by clinico-physiological and laboratory findings that supported its involvement (*14*). A specific interest in COVID-19 stems from features of endothelial injury and hypercoagulability, given the cross-talk between the complement and coagulation systems (*18*). This observation has resulted in multiple phase II and III clinical trials targeting various components of the complement system (*27*–*31*). However, many cytokines that have been implicated in COVID-19, are also elevated in other forms of acute infection, including those leading to respiratory failure (*6*, *32*). In certain instances, the levels of these cytokines in COVID-19 were lower than what was seen in these other diseases (*33*, *34*). Most studies to date on the role of the complement system in COVID-19 have not included a control group of patients with another infection, or with acute respiratory failure, as a result of which it has been unclear whether complement activation is a feature of COVID-19, or is a broader indicator of critical illness. Additionally, despite multiple in vitro lines of evidence, it is unclear which specific components of the system may be associated with worse outcomes in humans with COVID-19 in vivo, which has implications for appropriately targeting this system. In this manuscript, we demonstrate that: (1) markers of complement activation are higher in severe COVID-19, compared to those hospitalized with influenza or other forms of acute respiratory failure; (2) markers of complement activation distinguish those with worse outcomes in the setting of COVID-19, in two independent cohorts; (3) the alternative pathway is activated in patients with COVID-19, and is implicated in these worse outcomes; and, (4) components of the alternative pathway associate with markers of endothelial injury and increased coagulation, which are the clinico-physiological hallmarks of severe COVID-19 vasculopathy.

We observed that markers of complement activation are significantly higher in patients hospitalized with COVID-19, compared to those hospitalized with influenza or other forms of acute respiratory failure. Although complement activation can occur via convertase-dependent or convertase-independent pathways (e.g., thrombin cleaving C5 to C5a) in inflammatory settings such as lung injury and/or sepsis (*35*), multiple direct interactions between coronaviruses and the complement system may partly explain the elevated levels of these markers in patients with COVID-19 compared to the other etiologies. For example, the SARS-Co-V spike protein can bind to mannose-binding lectin (MBL) via an N-linked glycosylation site (*36*), initiating complement activation through the lectin pathway. The SARS-CoV-2 spike protein (subunits 1 and 2) has been shown to activate the alternative pathway in an in vitro system (*22*). Preliminary data point to the N-protein of SARS-CoV-2 mediating MASP2-drived complement activation (*21*, *37*). In comparison, the interactions between the influenza A virus (IAV) and components of the complement system appear to be more complicated. Although multiple models have demonstrated that complement activation occurs in influenza, IAV also evades complement by blocking the classical complement pathway through the M1 protein interacting with C1qA (*38*). Additionally, microthrombi are not as common in influenza as in COVID-19, and markers of hypercoagulability appear to be higher in COVID-19 (*12*, *13*). These may account for some of the reasons as to why complement activation is more pronounced in COVID-19, as compared to other etiologies of acute respiratory failure, including influenza.

In two independent cohorts, we demonstrate that markers of complement activation distinguish those who had worse outcomes in the setting of SARS-CoV-2 infection. The endothelial injury in COVID-19, especially in severe cases, has similarities to that seen in other forms of thrombotic microangiopathies, such as thrombotic thrombocytopenic purpura (*14*, *39*). In thrombotic microangiopathies, often a genetic predisposition, in combination with an inciting factor for endothelial damage, triggers a feed-forward loop which contributes to thrombosis and ongoing tissue injury (*14*). To that accord, a genetic predisposition toward complement activation has been reported in COVID-19 (*17*, *19*). Vis-à-vis markers of complement activation, C5a, generated from complement activation, is a potent chemoattractant for myeloid cells such as neutrophils and monocytes/macrophages (*40*). As myeloid cell activation is involved in the development of severe COVID-19 (*41*), one possibility is that C5a contributes to the overexuberant and likely pathogenic recruitment and activation of neutrophils and monocytes/macrophages (*24*). These infiltrating myeloid cells both express and release various complement components, and have anaphylatoxin receptors (i.e., C3aR, C5aR1) on their surfaces, that can bind to activated complement components (i.e., C3a, C5a), setting up putative autocrine amplification loops that have been reported in multiple cell types (*42*–*45*). Additionally, infiltrating neutrophils can express prothrombotic proteins such as tissue factor (TF), both via direct expression and via neutrophil extracellular traps, driving platelet-mediated NET-driven thrombogenicity (*16*). Amplifying the complement, clotting and coagulation cascades likely contributes to severe outcomes, such as acute respiratory failure needing admission to the intensive care unit, invasive mechanical ventilation, and death in certain cases (*24*).

A notable finding in our cohort is that components of the alternative pathway are increased in COVID-19. Alternative pathway activation has been implicated in COVID-19 pathogenesis using an in vitro system (*22*). Specifically, SARS-CoV-2 spike protein has been shown to directly activate the alternative pathway, and complement-mediated killing, as well as C3c and C5b-9 deposition on TF1PIGAnull target cells, was reduced by inhibition of Factor D (*22*). Transcriptomic analyses demonstrate that components of the alternative pathway (e.g., Factor B) are differentially increased in normal human bronchial epithelial (NHBE) cells infected with SARS-CoV-2 in comparison to NHBE cells infected with other respiratory infections, namely, respiratory syncytial virus, influenza (H1N1), and rhinovirus (RV16) (*4*). Additionally, increased serum levels of Factor B have been identified using a high-throughput screen of patients with clinically severe COVID-19 (*46*). In addition to components of the complement cascade being increased in human plasma, Factor D has also been reported as being up-regulated in monocytes of patients with COVID-19 pneumonia (*47*). We now show that not only is the alternative pathway activated in vivo, but Factor D also strongly correlates with markers of endothelial injury and increased coagulation in COVID-19, which are characteristic of severe disease. An important consideration is that the differences in AP activation were more profound than the absolute differences in the levels of Factor D. This difference also highlights redundancy in how Factor D activates the alternative pathway, an observation that has been reported but remains to be fully deciphered (*48*). This redundancy has also been recently observed in COVID-19, wherein SARS-CoV-2 was found to up-regulate local components of the alternative pathway in airway epithelial cells, but not Factor D (*49*). These observations suggest there may be serine proteases along with Factor D that can activate the alternative pathway in critical illness (*50*, *51*). Thus, Factor D-dependent compared to Factor D-independent activation of the alternative pathway in the setting of infection continues to be a field being actively explored by multiple groups, including ours. The next steps will be to understand the mechanistic basis for how SARS-CoV-2 activates the alternative pathway, and to evaluate whether interrupting alternative pathway-mediated activation could mitigate this vicious cycle that perpetuates tissue injury, at least in a subset of patients with severe COVID-19 who have this phenotype.

Our findings have several limitations. First, the samples were not simultaneously collected among the COVID-19, influenza, and non-COVID acute respiratory failure groups. However, they were collected in a similar time frame leading up to the SARS-CoV-2 pandemic, and subsequently processed using the same protocol, to minimize any differences in the findings. Second, we did not have levels of SARS-CoV-2 RNA to evaluate how complement activation correlates with viral load in COVID-19. A prior report suggested that markers of complement activation do not correlate with concurrently measured viral load (*15*). This is possible, given that the patients enrolled in our study are likely presenting in the “hyperinflammatory phase” of the illness, when viral loads may be lower than the initial phase of infection (*52*). Our next steps include exploring the mechanistic basis of how the alternative pathway is activated in endothelial cells, including the use of spike antigen assays. Third, decision-making in our ICU changed over a period of time; initially, there was a tendency for early intubation. Hence, we also included hospitalization and ICU admission in our data points; and provided data on mortality where applicable, derived from our electronic medical records. Fourth, for certain outcomes, our sample size was such that there were differences in the levels of the markers between the two groups, but they did not always meet statistical significance (e.g., mortality signal in sC5b-9); one explanation is that our study was not specifically powered for that outcome, and additionally, there may be other factors outside of acute respiratory failure that contributed to a signal such as mortality. Fifth, we observed lower values for complement analytes in the Yale cohort compared to the WUSM cohort. One explanation for this observation is that the values for markers of complement activation can be lower in plasma collected in citrate tubes compared to EDTA tubes (*53*). It is possible in the setting of an acute infection, this difference may be amplified. Other than this difference in anticoagulants, the samples were stored and processed similarly, keeping in mind the caveat that sample handling can affect measures of complement activation (*53*). Another explanation is that the samples were quantified on different instruments, although this should not be the primary reason for the difference. Finally, subjects in the WUSM cohort were sicker than those in the Yale (longitudinal) cohort because of which we report data from another Yale (cross-sectional) cohort of a higher severity. However, the differences observed in outcomes between the two cohorts suggest that increased complement activation remains a marker of adverse outcomes in COVID-19. The cutoff needed to establish which subjects will do worse, or need treatment, is being established in follow-up studies. Additionally, as we and others have previously reported, smaller differences in circulating proteins, especially in the context of complement activation, become apparent when studied locally (*54*, *55*). Reports on local complement deposition in autopsy specimens from patients with COVID-19 support this hypothesis (*9*, *10*, *26*). We did not have adequate bronchoalveolar lavage specimens to interrogate these differences; however, this is an area of active study in our laboratory.

In summary, we show that complement activation is greater in patients hospitalized with COVID-19 when compared to those with influenza or other forms of non-COVID acute respiratory failure. Certain markers of complement activation are associated with worse outcomes, including the increased risk of ICU admission and the need for invasive mechanical ventilation in patients with COVID-19. The alternative pathway is activated in these patients, and correlates with markers of endothelial injury and increased coagulation, which are characteristics of severe COVID-19. Although we demonstrate that increased activation of the alternative pathway is associated with worse outcomes in COVID-19, it remains to be determined whether it would be an optimal target in this disease, given the multiple mechanisms for its activation, especially in the context of acute lung injury (*50*, *56*). Moreover, although we have identified alternative pathway activation as a marker for worse outcomes in COVID-19; this does not exclude classical or lectin pathway involvement. Hence, much work remains to be done to better understand how and when to target the complement cascade, with the goal of mitigating disease severity due to SARS-CoV-2.

## MATERIALS AND METHODS

### Study Design

This was a prospective cohort study, which utilized plasma samples that had been independently collected from adults (aged ≥ 18 years) at two centers, Washington University School of Medicine and Yale School of Medicine. BRISQ reporting guidelines were followed and are reported in **Table S1** (*57*). The objectives of this study were to: (1) assess complement activation in cohorts of patients with acute respiratory failure due to COVID-19, and compare this activation to non-COVID acute respiratory infection, and acute respiratory failure needing invasive mechanical ventilation; (2) discern markers of complement activation that are associated with adverse outcomes in setting of SARS-CoV-2 infection; and (3) identify the pathways by which the complement cascade was activated in patients with COVID-19.

### Research Subjects

At Washington University School of Medicine, we included plasma samples from patients presenting to the hospital with COVID-19 between March 26, 2020 to May 9, 2020 (‘WUSM cohort’) (*6*). Diagnosis of COVID-19 was based on a positive nasopharyngeal swab test. Inclusion criteria required that patients be symptomatic and have a physician-ordered SARS-CoV-2 nasopharyngeal swab test performed in the course of their normal clinical care. The first available sample from the patient was utilized for analysis, primarily within 24 hours of hospital admission. 95% (127/134) of samples assessed for sC5b-9 at WUSM were collected within 24 hours of admission. The other 7 samples were included because they were the earliest available samples for those patients in this cohort. Even after excluding these 7 samples, sC5b-9 levels were significantly higher in those patients needing invasive mechanical ventilation (p=0.025). All samples in which Factor B, Ba, C5a and Factor D were assessed, were collected within 24 hours of admission. Other clinically relevant medical information was collected at the time of enrollment from the patient, their legally authorized representative, or the medical record.

We also report findings from influenza-infected patients enrolled in separate, ongoing studies (i.e., EDFLU study) (*58*). These patients were sampled between 2017-2020, although most were enrolled during the 2019 to 2020 influenza season, prior to the spread of COVID-19 in the St. Louis region.

To have a comparable cohort of patients with non-COVID acute respiratory failure requiring invasive mechanical ventilation, we utilized samples from the ongoing IPS (Immunity in Pneumonia and Sepsis) study at WUSM. These samples were also collected from 2019-2020 among patients admitted to the ICU, on mechanical ventilation, prior to the spread of COVID-19 in the Saint Louis region.

At Yale School of Medicine, plasma samples from 23 patients with COVID-19 were collected between April 13, 2020 to April 24, 2020 (‘Yale longitudinal cohort’) (*59*, *60*). A second Yale cohort (‘Yale cross-sectional cohort’) was also analyzed, which included blood samples obtained either on day 1 (within 24 hours), day 4, and/or day 7 of hospitalization from 49 consecutive adult patients who were admitted for treatment of laboratory-confirmed COVID-19 between May 23, 2020 and May 28, 2020 and remained hospitalized until at least day 4. Diagnosis of COVID-19 was based on a positive nasopharyngeal swab test using PCR assays. Inclusion criteria required that patients be hospitalized and had a physician-ordered SARS-CoV-2 nasopharyngeal swab test performed in the course of their normal clinical care.

### Sample Size

We included consecutively collected plasma specimens from each cohort that were made available to us. Thus, the numbers were not altered during the course of the study. In certain cases (e.g., multiplex complement analytes), a subset were chosen based on sample availability.

### Outcome Definition

The prospectively selected primary outcome was the need for ICU admission. Secondary outcomes included the need for invasive mechanical ventilation (IMV), and 28-day mortality. These outcomes were abstracted utilizing an honest broker system from electronic medical records.

### Sample Collection and Processing

The processing of the samples in the laboratory was similar among the cohorts. Analytes were measured in cell-free plasma collected from patients within the first 24 hours of emergency department presentation. In the WUSM COVID-19 and influenza cohorts, blood samples were collected in EDTA-containing vacutainers (BD Biosciences, San Jose, CA), transported on ice and spun down at 2,500 g (4,725 rpm) for 10 min at 4°C, after which they were stored at −80°C until further analysis (*6*, *61*).

In the WUSM non-COVID (IPS) cohort, blood samples were collected in EDTA-containing vacutainers (BD Biosciences, San Jose, CA), transported on ice and spun down at 3,500 rpm for 10 min at 4°C, after which they were stored at −80°C until further analysis.

In the Yale COVID-19 cohorts, due to diurnal variations in certain analytes (e.g., plasminogen activator inhibitor-1 (PAI-1)), for hospitalized patients, blood specimens were collected with the first scheduled morning draw (i.e., occurred between 0300 hours and 0700 hours). For measurements of complement, coagulation and endothelial cell markers, blood was collected in 3·2% sodium citrate tubes and centrifuged at 4000 rpm at room temperature for 20 min. The resulting plasma supernatant was used for further testing. All of our samples were run in duplicate, but at two independent centers (WUSM and Yale). At both centers, the plasma samples were stored at -80°C between collection/plasma separation and analysis. Our measurements involved analyzing 38-40 samples at a time per plate, and the values were extrapolated from a standard curve. For each plate, a separate standard curve was performed, which enhances accuracy. The same batch (lot number) of assays were used at each individual center, but were likely to be different between the two centers. Further details on processing the samples have been reported in prior publications (*59*, *60*).

### Measurements of complement components

Details regarding the assays, location where they were performed, and the kits and instruments used for analysis have been provided in Table S2 (*23*, *62*, *63*).

### a) Soluble C5b-9 assay

Participants were screened in duplicate for complement activation in the plasma using the soluble C5b-9 (sC5b-9) assay (BD OptEIA Human C5b-9 ELISA set, Franklin Lakes, NJ, USA) (*54*). Per the manufacturer, purified native human C3, C4, C5, C6, C7, C8 and C9 were tested in the BD OptEIA assay at ≥ 5 mg/ml and no cross-reactivity (value ≥ 470 pg/ml) was identified.

### b) Individual complement analytes

Individual complement analytes were evaluated in duplicate using a modified MILLIPLEX MAP Human Complement Panel 1 and 2 (MilliporeSigma, Burlington, MA, USA) based on the Luminex xMAP technology, a bead-based multiplex assay. Specifically, we employed the MILLIPLEX MAP Human Complement Panel 1 kit (HCMP1MAG) to simultaneously quantify the following analytes in the plasma: C5, C5a and Factor D. The C5 measurements are distinct from C5a, as the intact factor assays are designed such that they would not detect individual fragments based on their capture and/or detection antibodies. Similarly, the C5a assay (beads) do not cross-react with C5, per discussion with the manufacturer. We also used the MILLIPLEX MAP Human Complement Panel 2 kit (HCMP2MAG) to simultaneously quantify the following analytes in the plasma: C3, C3b/iC3b, and Factor B. Of note, the assay for C3b/iC3b detects both C3b and iC3b (per communication with manufacturer). The assay for Factor B in this kit does not detect either fragment Ba or Bb. For both these assays (HCMP1MAG and HCMP2MAG), both intra- and inter-assay precision was < 10% coefficient of variance (CV). Intra-assay precision was generated from the mean of the %CV from 8 reportable results across two different concentrations of analytes in a single assay. Inter-assay precision is generated from the mean of the %CV across two different concentrations of analytes across 8 different assays.

### c) Alternative pathway (AP) analytes

Ba was measured in duplicate in the WUSM COVID-19 cohort using the MicroVue Complement Ba fragment EIA kit (A033, Quidel Inc,San Diego, CA, USA). For the Ba assay, intra-assay precision was 2.2-3.3% and inter-assay precision was 2.4-8.1%. AP hemolysis assays were performed using a rabbit red blood cell (RBC) assay for AP activity of the plasma samples. 1 microliter of rabbit RBCs was incubated in AP buffer (gelatin veronal buffer with 20 mM MgCl_2_ and 8 mM EGTA) with 10% sample concentration for 1 hour. Released hemoglobin was measured at an optical density (O.D.) of 405 nm. Lysis of rabbit RBCs in water served as the positive control whereas rabbit RBCs in AP buffer served as the negative control. Hemolysis percentages were determined by an O.D. ratio using the following formula: (10% plasma with RBC in AP buffer - 10% plasma without RBC in AP buffer)/(RBC in water - RBC in AP buffer).

### d) Measures of coagulation and endothelial cell markers

vWF:Ag was measured at the Yale New-Haven Hospital (YNHH) Clinical Laboratory using ACL TOP (Instrumentation Laboratory; Bedford, MA, USA) with manufacturer’s reagents and controls per laboratory protocol using a latex enhanced immunoassay. The vWF:Ag assay used polystyrene particles coated with rabbit polyclonal antibody directed against vWF:Ag. The results are reported as percentages compared with calibration curves using values obtained from the standardized reference population used for clinical laboratory testing throughout the YNHH system. Soluble thrombomodulin was measured using ELISA assays (Abcam, ab46508), wherein samples were diluted in a 1:4 ratio before addition to ELISA plates. Ang2 plasma levels were measured by Eve Technologies (Calgary, Alberta, Canada). Assays were done in duplicate according to the manufacturer’s instructions.

### Blinding

Investigators running the assays were blinded to the clinical outcomes of the patients under consideration, or the subgroups being compared.

### Statistical Analyses

Based on normality testing, we used non-parametric tests for comparison. Specifically, two independent groups were compared using the Mann-Whitney U test. Statistical tests for comparison were two-sided, and p < 0.05 was considered significant. In the violin plots, the central line in the plot represents the median, the two lines on either side of the median represent the interquartile range, while the length denotes the distribution. All outliers were included in the data. The correlation between complement activation proteins and measures of endothelial injury and hypercoagulability was assessed using Spearman’s rank correlation, and plotted utilizing a simple linear regression line, with the error bars denoting the 95% confidence intervals. Statistical analysis was performed using IBM SPSS Statistics for Macintosh, Version 27.0 (IBM Corp, Armonk, NY), and GraphPad Prism 9 (GraphPad Software, La Jolla, CA) was employed for generating figures.

### Study Approval

The local Institutional Review Board approved this study at both Washington University School of Medicine (ID#201707160, 201801209, 201808171, 201710220, 201808115, and 201910011, 201904191, 202004091, 202003085) and independently at the Yale School of Medicine (IRB 2000027792 and 1401013259).

## Supplementary Materials

immunology.sciencemag.org/cgi/content/full/6/59/eabh2259/DC1 Fig. S1. Markers of complement activation are higher in COVID-19 compared to non-COVID-19 respiratory failure. Fig. S2. Complement activation is associated with worse outcomes in patients with severe COVID-19. Fig. S3. Components of the alternative pathway are associated with worse outcomes in COVID-19. Table S1. Biospecimen reporting for improved study quality (BRISQ) Reporting Criteria. Table S2. Normal values and methodology for quantifying complement analytes in plasma samples at WUSM and Yale School of Medicine. Data file S1. Raw data file (Excel spreadsheet)
